# Case of Sloughing Ulcer—Amputation—Death from Phlebitis

**Published:** 1838-09-26

**Authors:** A. M. Vedder

**Affiliations:** Resident Surgeon


### PHILADELPHIA HOSPITAL.

### *Case of Sloughing Ulcer—Amputation—death from Phlebitis.*

[Reported by Dr. A. M. Vedder, Resident Surgeon.]

Dennis O'Leary, aged 36 years, born in Ireland, in America for ten years, an intemperate labourer, was admitted into the hospital, for sloughing ulcer. He has had an attack of intermittent fever, almost yearly, since he has been in this country. About five years since he had an attack in Philadelphia, continuing for nineteen months; it was, at first, of the quotidian type, and treated usually with quinine. Six or eight years since, while splitting wood, he was cut on the shin with a wedge. After a long time, the wound healed up, breaking out at times afterwards; it has never been entirely closed for the last three years. He had pain in it at times. Says he had dropsy a year since; his inferior extremities were swollen to twice their natural size; abdomen distended. He has been salivated twice, the last time in July, 1838. Says he never had jaundice. In July last, he was taken with singultus, which continued five days; his ulcer was sloughing at the same time; never lost his appetite. A second attack of singultus came on about the 1st of August, continuing incessantly for two weeks, the ulcer having again commenced sloughing. Stimulants, carminatives, tonics, and a generous diet were given.

At the time of his admission, his condition was the following:—

Complexion yellow, conjunctiva partaking of the same colour; moderate emaciation; tongue rather dry; appetite moderate; thirst considerable; slept well; intelligence clear; skin of natural temperature; pulse, seventy-eight, of moderate strength and fulness. A large sloughing ulcer occupied the middle of the tibia of the right extremity, exposing the tibia for the space of seven inches. About two-thirds of the circumference of the limb were involved; very offensive odour; complained of pain in the part; right foot cooler than the left, and edematous; a sloughing ulcer, about two inches in diameter, existed on the tibia of the left limb; the man was very anxious to have the limb amputated.

*August 23d.*—A consultation of the visiting surgeons having been called, it was determined to amputate the limb. Dr. Pancoast, in the presence of Dr. Harlan, the resident physicians, and several students, amputated the limb, just below the knee, sufficiently low to avoid the attachment of the patella; very little blood was lost. The circular operation was performed, and the flaps brought together after the French manner. The patient bore the operation well; ordered porter, brandy, and beef essence.

*August 28th.*—The weather being cool, the dressings were not removed until this morning. Stump discharged very little; the flaps had not united. Since the operation he said he suffered less pain than previously. Slept well; appetite improved; strength about the same; ulcer on the other limb improved. Continued treatment. T. Cinchon. c. f℥i., quâque 2d âhorâ.

*August 30th.*—Severe chill this morning, lasting about ten minutes, followed by fever. Delirious on the night of the 29th. Appetite less. Thirst great; slight cough; no expectoration. Skin hot and dry, (in the axilla one hundred and four degrees.) Pulse one hundred and sixteen, quick. Respiration thirty. The stump presented an unhealthy appearance; gangrenous odour; an ulcer of the size of a shilling on the flap; tongue rather dry and yellow. Continued treatment. Poultice to the stump.

*September 2d.*—Countenance fixed; respiration high and frequent; rattling in the throat; abdomen much distended; tympanitis; evidently moribund. The man was sensible; died the same morning.

Autopsy, twenty-four hours after death.

Emaciation considerably advanced. Skin of a light saffron colour. *Thorax:* a few ounces of reddish serum in both pleuræ. The lower lobe of the right lung congested, contained air; a reddish spumous fluid ran out from the cut surface, when pressed; tissue more easily torn than common. The upper and middle lobes were distended with air, and contained some fluid; no tubercles; tissue firmer than that of the lower lobe; bronchial tubes pale. Left lung. The lower lobe was very similar, in texture and appearance, to the same lobe of the right side; the tissue was more friable, however, than the right; upper lobe distended with air, of a light gray colour externally; no tubercles; bronchial tubes natural.

Liver, voluminous, thirteen inches in length, eight inches in breadth, and four inches in depth; the superior, two-thirds were of a black colour; texture rather spongy; very friable, breaking down on the least pressure; the remainder of a brown colour, and more firm in its texture. Gall bladder pale externally; contained half an ounce of greenish bile. Spleen eleven inches in length, seven inches in breadth, and three and a half in depth. It was of an irregular shape; soft and elastic. External membrane thickened. On making an incision longitudinally, through the centre, the tissue was found to be pulpy, almost diffluent, of rather a bright red colour; weight estimated at four or five pounds. Heart rather small; tissue softened; the internal membrane of the left ventricle tinged, and that of the left presented the usual appearance; all the valves were soft and flexible. The aorta, laid open in different parts, was of a light yellow colour; the internal membrane smooth. The internal membrane of the vena cava ascendens was tinged red, the surface smooth, not thickened; the contained blood was fluid, and of a brown colour. The iliac veins, on both sides, presented the same appearance. The vein accompanying the femoral artery, was, at first, mistaken for the artery itself, on account of the thickness and firmness of its coats, but it was recognised by the valves. It contained some firm fibrous coagula, and brown fluid blood. The internal coat was thickened, say as thick as writing paper, its colour was blue; the other coats were also much thickened, thicker, indeed, than the coats of the aorta; the tissue surrounding the vessel was hard and firm; the inferior six inches of the vein were found to be filled with a soft yellow purulent matter, the membrane beneath opaque. The artery was tinged and thickened, more marked as we approached the stump; its extremity was filled with a coagulum. The artery and vein of the other extremity were natural.

Some doubt was entertained of the propriety of the operation, on account of the state of the patient's constitution. Dr. Pancoast performed it with reluctance. The man's death was certain without the operation, and it gave him a small chance. The hypertrophy of the spleen, in connexion with the anterior history, offers two interesting points, namely, the connexion between intermittent fever and hypertrophy of this organ, the dropsy following, as the consequence of the latter. The immediate cause of death was, probably, phlebitis, which was developed three days before the man's decease. The lungs were just in that condition in which pus is deposited by metastasis, and if the patient had lived a day or two longer, pus would probably have been deposited in these *organs.*

